# A Simple Doping Process Achieved by Modifying the Passivation Layer for Self-Aligned Top-Gate In-Ga-Zn-O Thin-Film Transistors at 200 °C

**DOI:** 10.3390/nano12224021

**Published:** 2022-11-16

**Authors:** Cong Peng, Huixue Huang, Meng Xu, Longlong Chen, Xifeng Li, Jianhua Zhang

**Affiliations:** Key Laboratory of Advanced Display and System Applications of Ministry of Education, Shanghai University, Shanghai 200072, China

**Keywords:** InGaZnO, self-aligned top-gate, thin-film transistors

## Abstract

In this paper, a facile modifying technique of source/drain regions conductivity was proposed for self-aligned top-gate In-Ga-Zn-O (IGZO) thin-film transistors (TFTs) by controlling the process parameter of the passivation layer at relatively low temperatures. The sheet resistance of the source and drain regions of IGZO was approximately 365 Ω/□, and there was no significant change within a month. The device parameters of mobility, threshold voltage, subthreshold swing, and current switching ratio of the fabricated device were 15.15 cm^2^V^−1^s^−1^, 0.09 V, 0.15 V/dec, and higher than 10^9^, respectively. The threshold voltage drift under negative bias illumination stress was −0.34 V. In addition, a lower channel width-normalized contact resistance of 9.86 Ω·cm was obtained.

## 1. Introduction

Nowadays, amorphous indium gallium zinc oxide (a-IGZO) receives significant attention as an active layer of thin film transistors (TFTs) in the display industry because of its high mobility, very low off-state current, low process temperature, and intermittent refreshing [1,2,3,4,5,6,7]. The typical bottom gate structure of AOS TFTs, including back-channel-etch and etch-stopper-layer, inevitably suffer from resistor–capacitor delay and image lagging, which is mainly due to the parasitic capacitance formed by the overlap between the gate (G)-source/drain (S/D) electrodes [8]. However, the source/drain and gate of the self-aligned top gate (SATG) structure are located on the same side of the active layer and have relatively small parasitic capacitance, so they have drawn the attention of more and more researchers [5,9,10,11,12,13,14]. The key technical challenge of SATG TFT is how to form source–drain regions with low resistance. Up to now, several solutions have been put forward to rise to this challenge, such as ion implantations [9], metal reaction-induced [5,10], plasma treatment [11], and hydrogen (H) doping in time of interlayer dielectric or passivation deposition [12,13]. Nevertheless, some problems with these methods cannot be ignored. Regarding the high activation temperature of ion implantations, guaranteed precise film thickness with metal reaction-induced, plasma-induced damages effect the plasma as a result of the uncontrollable rapid hydrogen diffusion of the high-temperature activation process with hydrogen incorporation. Furthermore, low-resistance IGZO films can also be prepared by coating with organic interlayer dielectric [15] or ultraviolet irradiation [16], which may result in an undercut and worse step coverage due to the gate stack directly formed by one-step dry etching. Based on the key technical challenges of SATG TFTs, there are currently few reports on the indirect processing of IGZO source–drain regions to form highly conductive regions in active oxide channels. Thus, in this study, we propose the vertical diffusion of hydrogen by modifying the hydrogen content of the passivation layer process to form low-resistance IGZO source–drain regions for indirectly realizing self-aligns. This simple doping process not only optimizes the process steps for reducing the resistance of the source/drain regions but also improves the water and oxygen barrier capabilities of the device. SATG TFTs with good electrical properties and excellent negative bias illumination stress (NBIS) stability were successfully fabricated under a low-temperature process.

## 2. Materials and Methods

**Figure 1:**
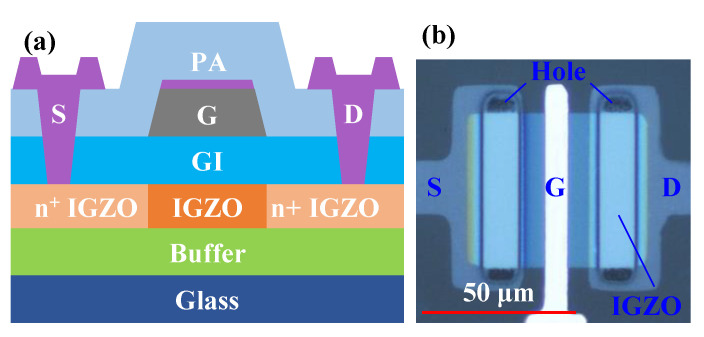
(**a**) The schematic diagram of SATG IGZO TFTs. (**b**) Top view of SATG IGZO TFT.

First of all, a 200 nm thick buffer layer with SiO_2_ was deposited on 200 × 200 mm glass by plasma-enhanced chemical vapor deposition (PECVD). Then, the IGZO with a thickness of 40 nm was sputtered as an active layer and was patterned using a wet etchant. Furthermore, SiO_2_ (SiH_4_/N_2_O = 6/94) with the thickness of 300 nm was deposited as a gate insulating layer (GI) by PECVD at 200 °C. Secondly, Mo (30 nm)/ITO (5 nm) was sputtered as a gate electrode using magnetron sputtering. Next, the gate electrode was formed by a two-step wet etch of the Mo/ITO stack. After SiO_2_ (SiH_4_/N_2_O = 6/94) or Si_3_N_4_ (SiH_4_/NH_3_/N_2_ = 6/22/72) with a thickness of 200 nm was deposited as a passivation layer (PA) at 200 °C., the n^+^ IGZO extension regions (the size of one side is 25 × 50 μm^2^) were automatically formed through the gate. Moreover, the contact holes were exposed by dry etching. In the end, the ITO with a thickness of 35 nm was sputtered again by sputtering as a source–drain electrode. It is worth noting that the process temperature during device fabrication was not higher than 200 °C. The channel width of the device was fixed at 50 μm, and the channel length (*L*) was 4 μm, 6 μm, and 8 μm, respectively. Figure 1a,b represents the schematic cross-section and top optical image of the SATG IGZO TFT, respectively.

The electrical characteristics for the fabricated a-IGZO TFTs were measured using Keithley 4200. We define the corresponding gate voltage (*V_GS_*) when the leakage current (*I*_DS_) is 1 nA as the threshold voltage (*V_TH_*) of the TFT device. The mobility (*μ*) was calculated according to the following equation: *μ* = 2*L*·*I_DS_*/*W*·*C_i_*·(*V_GS_*−*V_TH_*)^2^, where *C_i_* is the gate of the capacitance per unit area. The sheet resistance was measured by the 3 m mini type four-probe tester in the dark at 300 K. The light source used in the NBIS tests was white light with a brightness of 10,000 lux by applying *V_G-Stress_* = −10 V of 1000 s with source and drain electrodes grounded. The light source was emitted from the bottom of the device.

## 3. Results and Discussion

To verify the effect of the passivation layer process with different hydrogen contents on device characteristics, SATG IGZO TFTs with three passivation layers were fabricated without a passivation layer (*w*/*o*). SiO_2_ and Si_3_N_4_, respectively, represent the low, medium, and high hydrogen content in the passivation layer film.

**Figure 2:**
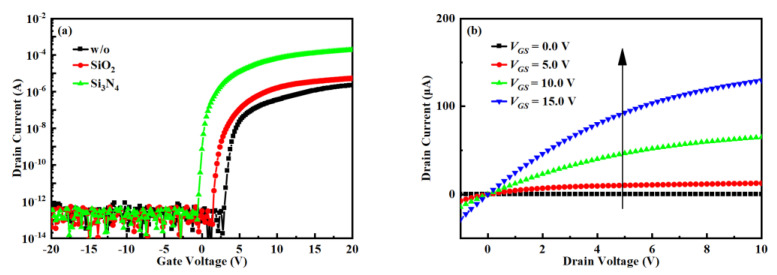
(**a**) Variation of *I*_DS_-*V*_GS_ transfer curves of SATG IGZO TFTs prepared with different passivation layers, (**b**) output curve of SATG IGZO TFT with Si_3_N_4_ as a passivation layer.

The transfer performance with SATG IGZO TFTs with different passivation is shown in Figure 2a. When the hydrogen concentration of the passivation layer increases, the *μ* increases from 0.31 to 15.15 cm^2^V^−1^s^−1^, threshold voltage (*V_TH_*) from 3.91 V to 0.09 V, subthreshold swing (*SS*) from 0.10 V/dec to 0.15 V/dec, and *I*_on_/*I*_off_ increases from 1.17 × 10^8^ to 2.86 × 10^9^. Figure 2b show the output characteristic of TFT with Si_3_N_4_ as passivation. The output characteristics show good ohmic contact at low drain voltages and low source/drain resistance [17]. This can be attributed to not only higher electron mobility but also lower contact resistance [10,18].

It can be intuitively seen from Figure 2a that the *I*_on_ of the device gradually increases from ~10^6^ A to ~10^4^ A as the hydrogen content increases. Although the IGZO/GI interface will have a serious impact on *I*_on_, the fabrication processes of the three devices are basically the same, that is, the IGZO/GI interface defects should be the same, which can be reflected from the *SS* value with no obvious change. Therefore, the increase of *I*_on_ is mainly due to the decrease of the contact resistance. After the passivation layer is deposited, since the passivation layer contains a high concentration of hydrogen, a large amount of hydrogen will first pass through the insulating layer from the passivation layer and then vertically diffuse to the IGZO layer [19]. In addition, because the densified gate can block the diffusion of hydrogen, hydrogen will vertically diffuse down to the IGZO layer along the edge of the gate, reducing the resistance and self-aligning to form the IGZO source and drain regions. Although a small amount of hydrogen will diffuse laterally, it will only remain in the insulating layer above the channel. As the hydrogen content in the passivation layer increases, the hydrogen diffused into the IGZO layer must increase, so the formed IGZO source and drain regions have better conductivity, and the TFT device has smaller parasitic resistance. At the same time, the channel carrier concentration increases, and the V_TH_ shifts to the left.

**Figure 3:**
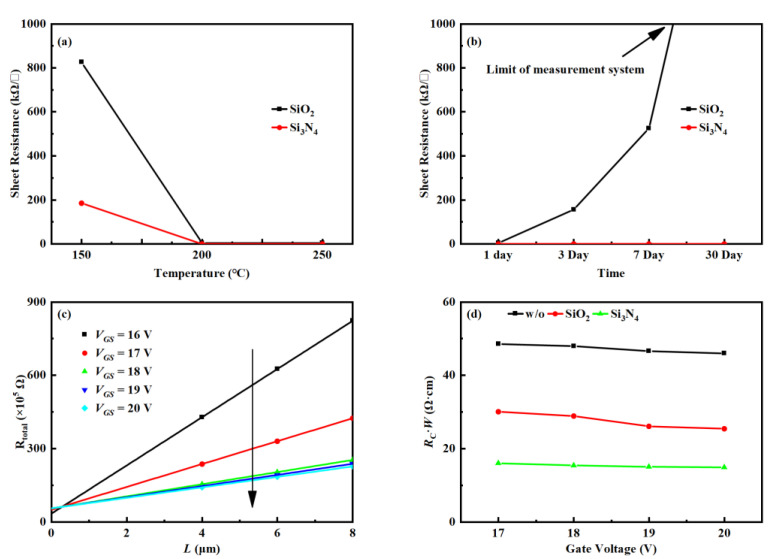
(**a**) Variation of sheet resistance with deposition temperature, (**b**) variation of sheet resistance with placement time, (**c**) *R_tot_-L* plot measured from the TFTs with different *L* at various gate voltage biases, (**d**) width-normalized contact resistance as a function of gate voltage.

To demonstrate that the contact resistance is reduced, the sheet resistance of S/D regions in the IGZO was measured. Figure 3a show the variation of sheet resistance of the IGZO source and drain regions with deposition temperature. The sheet resistance decreases gradually with the increase of deposition temperature. At 150 °C, the sheet resistance corresponding to the Si_3_N_4_ passivation layer is much lower than that of SiO_2_, but when the temperature increases to 200 °C, the effects of the two passivation layers are similar and remain basically unchanged. The sheet resistance varies around 365 Ω/□. Therefore, its resistivity is 1.5 × 10^−3^ Ω·cm because its thickness is 40 nm. Table 1 summarize the resistance and device mobility obtained by different treatments for the source and drain regions of IGZO. In this work, the PA/GI processing approach exhibits low resistivity and high device mobility and achieves a level comparable to other methods.

As shown in Figure 3b, with the passage of time, the two passivation layers have different changes to the IGZO source and drain regions. The Si_3_N_4_ passivation layer maintained good stability for one month, while the SiO_2_ passivation layer showed poor stability. After 3 days, the sheet resistance increased to more than 156 kΩ/□, increased to about 526 kΩ/□ after a week, and exceeded the test limit of the device after a month. This can be attributed to the low hydrogen content in the SiO_2_ film, which has an insufficient degree of influence on the IGZO film. With the increase of the standing time, the hydrogen in the SiO_2_ film diffused outward, reducing the hydrogen content in the IGZO film, and thus the sheet resistance increased.

The contact resistance (*R*_C_) was calculated according to the transmission line method. It includes the contact resistance between the metal and semiconductor and the resistance of the S/D extension part inside the semiconductor. The following equation may be used to calculate the *R_tot_* [15]:

(1) $$R_{tot}=\frac{V_{DS}}{I_{DS}}=\frac{R_{S}}{W}L+2R_{C}$$

*R_S_* is the channel resistance. Figure 3c show the total resistance (*R_tot_*) corresponding to different *L* at various *V*_GS_. The transmission line method was used to extract the width-normalized *R*_C_ (*R_C_·W*) and the diffusion distance, as shown in Figure 3d [5]. For SATG IGZO TFT with Si_3_N_4_ passivation layer, the *R_C_·W* values were approximately 9.86 Ω·cm, and the lateral diffusion distance was only 0.07 μm.

**Figure 4:**
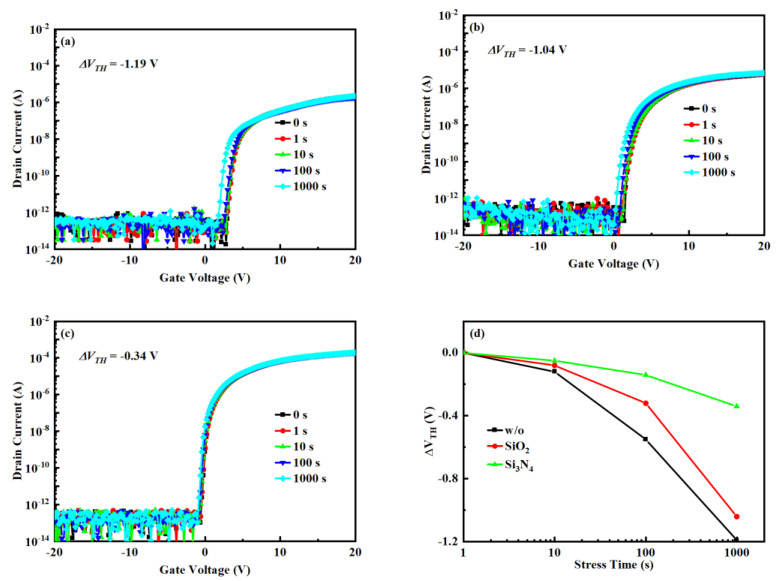
Evolutions of transfer characteristics of the IGZO TFTs with increasing NBIS: (**a**) *w*/*o*, (**b**) SiO_2_, (**c**) Si_3_N_4_, (**d**) Δ*V_TH_* as a function of stress time.

To verify the prospect of passivation in practical applications of SATG IGZO TFT, the NBIS is shown in Figure 4. As the time of illumination and gate voltage are increased, the V_TH_ drifts in the negative direction. However, the threshold voltage drift (Δ*V_TH_*) of TFTs with *w*/*o* or SiO_2_ is reduced from −1.19 V or −1.04 V to −0.34 V compared to TFTs with Si_3_N_4_. This shows that the Si_3_N_4_ can improve device stability. The V_TH_ drift under NBIS is mainly due to the formation of positively ionized oxygen vacancies under the combined effect of illumination and negative gate voltage (V_O_ → $V_{O}^{+}$/$V_{O}^{++}$) [20]. The *ΔV_TH_* for SATG IGZO TFTs with Si_3_N_4_ as passivation is as low as −0.32 V, which may be due to the reduction of positive charge ($V_{O}^{+}$/$V_{O}^{++}$) by the formation of substitutional hydrogen (H^O^ + e^−^ → H^−^ + V_O_ → H_O_), where H^O^ and H_O_ represent a hydrogen atom and substitutional hydrogen, respectively [21]. In addition, as the hydrogen in the passivation layer increases, the lateral diffusion of hydrogen remaining in the insulating layer also increases. In subsequent processes, hydrogen will diffuse from the GI to the IGZO/GI interface, passivate the V_O_–related point defects, and reduce the deep donor electron traps (H^O^ + O^2−^ → OH^−^ + e^−^) [4,20]. At the same time, hydrogen implantation from GI to the IGZO/GI interface also increases the carrier concentration and reduces the interfacial trap states, thereby reducing Δ*V_TH_* [22,23].

## 4. Conclusions

In this paper, SATG TFTs were successfully fabricated by modifying the hydrogen content of the passivation layer process under a low-temperature process. The influence of the passivation layer on the electrical properties was compared, and it was proven that the modification of the passivation layer process can effectively reduce the resistance of the source and drain regions of IGZO. The prepared TFT had a low *R_C_·W* of 9.86 Ω·cm and a low lateral diffusion distance of 0.07 μm. The fabricated SATG TFT exhibited excellent electrical properties with *μ* of 15.15 cm^2^V^−1^s^−1^ and *I*_on_/*I*_off_ higher than 10^9^, respectively. Meanwhile, NBIS stability was remarkably improved from −1.19 to −0.34 V.

## Figures and Tables

**Table 1:** This is a table. Tables should be placed in the main text near to the first time they are cited.

Treatment Methods	*μ* (cm^2^V^−1^s^−1^)	Resistivity (Ω·cm)	Temperature (℃)	References
PA/GI	15.15	1.5 × 10^−3^	200	This Work
Ion implantations	7	-	350	2021 [9]
Metal reaction-induced	13	2.4 × 10^−3^	200	2021 [10]
Plasma	5.13	2 × 10^−3^	350	2021 [11]
Zeocoat	18.84	3.8 × 10^−4^	150	2020 [15]
Interlayer dielectric layer	14	3 × 10^−3^	250	2019 [13]
ultraviolet irradiation	6.7	3 × 10^−5^	300	2015 [16]

## Data Availability

The data and contributions presented in the study are included in the article. Further inquiries can be directed to the corresponding author.
